# Design and Implementation of a Digital Angular Rate Sensor

**DOI:** 10.3390/s101109581

**Published:** 2010-10-28

**Authors:** Li-Feng Wu, Zhen Peng, Fu-Xue Zhang

**Affiliations:** 1 Beijing Engineering Research Center of High Reliable Embedded System, Capital Normal University, Beijing, 100048, China; 2 Department of Computer, North China Institute of Science and Technology, Beijing, 101601, China; E-Mail: zhen_peng1981@163.com; 3 Research Center of Sensor Technology, Beijing Information Science & Technology University, Beijing, 100101, China; E-Mail:zhangfuxue@263.net

**Keywords:** gyroscope, accelerometer, pitch, yaw, angular rate

## Abstract

With the aim of detecting the attitude of a rotating carrier, the paper presents a novel, digital angular rate sensor. The sensor consists of micro-sensing elements (gyroscope and accelerometer), signal processing circuit and micro-processor (DSP2812). The sensor has the feature of detecting three angular rates of a rotating carrier at the same time. The key techniques of the sensor, including sensing construction, sensing principles, and signal processing circuit design are presented. The test results show that the sensor can sense rolling, pitch and yaw angular rate at the same time and the measurement error of yaw (or pitch) angular rate and rolling rate of the rotating carrier is less than 0.5%.

## 1. Introduction

In recent years, MEMS sensors have become more and more popular in the industrial and automotive fields. Because the MEMS angular rate sensors have the characteristics of high performance, extremely compact size, low power operation and low cost, they has been considerable interest in their design and fabrication [1,2]. Currently, there are many micromechanical gyroscopes (angular rate sensors), including electrostatically driven [3,4], electromagnetic driven [5–7] and piezoelectric driven [8,9] ones, *etc*., designed to measure the angular rate or the rotation angle by integrating the measured angular rate with respect to time. These gyroscopes have drive parts and sensing parts, so their structures are complex. We are investigating a novel MEMS-based gyroscope, which has no driving parts, and utilizes the circumrotation of the rotating carrier itself as driving force. Therefore, it is suitable for detecting the angular rate of a rotating carrier due to its characteristics.

The design, fabrication and basic performance of the gyroscope have been reported in [10]. Moreover, we have shown that rotating carrier rotational velocity instability influences the output signal, and presented a method to eliminate the effect in actual applications [11]. Now, based on the sensing element, a novel digital angular rate sensor is proposed. It includes sensing elements and signal processing circuits. The digital angular rate sensor can sense three angular rates of a rotating carrier and output three digital angular rate signals.

## 2. The Sensing Element and Operating Principles

### 2.1. Sensing Element

**Figure 1.**
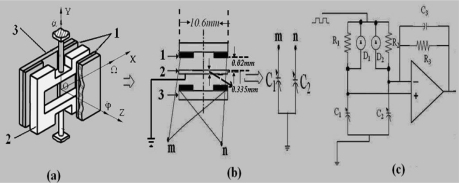
The structure and signal processing of the sensing element: **(a)** lateral view of the structure of sensing element, 1-the copper electrode, 2-the silicon pendulum, 3-the ceramic substrate; **(b)** the differential-capacitance; **(c)** the C/V transformed circuitry.

The structure of the sensing element is shown as Figure 1(a). It consists of a silicon four leaf clover structure. The silicon pendulum is obtained through bulk micromachining technology and attached to the center. It can form two pairs of capacitances with the opposite copper plating ceramic substrate, respectively.

The dimensions of the sensing element are illustrated in Figure 1(b). The silicon pendulum is connected with an analog ground, and the four pieces of copper plating ceramic substrate are connected together, as shown in Figure 1(b). Thus we can obtain a pair of differential capacitors, shown in Figure 1(b), right. Figure 1(c) is the corresponding C/V transformed circuitry. It is clear that the capacitance of these four capacitors varies with the angle velocity Ω. When the change of differential capacitance is small it is easily disturbed by the distributed capacitance, while in the pick-up circuit, the alternating-current bridge is used as the interface of the transfer circuit. The differential capacitance is discharged and charged by a square wave pulse current, then amplified by amplifier, finally the output voltage signal, which is directly proportional to the angle velocity Ω, is obtained.

### 2.2. Sensing Principle

The sensing principle of the sensing element is based on the measurement of Coriolis forces. From Figure 1(a), the sensing element is installed on a rotating carrier and rotates together with the rotating carrier along the OZ axis. Once the rotating carrier has a yaw or a pitch angular rate (along the OX or OY axis direction), the silicon pendulum swings around the internal sensitive OX axis under the Coriolis forces. Therefore, the differential capacitance C_1_, C_2_ can sense yaw or pitch angular rate. According to the C/V transformed circuitry, as shown in Figure 1(c), we can obtain:

(1) $$u_{o}=f(\dot{\phi })\Omega \text{sin}(\dot{\phi }t+\theta_{1}(t))$$

where *φ̇* is the rolling frequency of rotating carrier, Ω the fused yaw and pitch angular rate, *u_o_* the output voltage, *θ*_1_(*t*) the initial phase, *f*(*φ̇*) the transmission factor, which depends on the *φ̇*. In order to eliminate the effect of *φ̇*, according to the method [11], *u_o_* can be expressed as *u*_1_ as follows:

(2) $$u_{1}=k\Omega \text{sin}(\dot{\phi }t+\theta_{1}(t))$$

where *k* is the scale factor. From the function (2), it is clear that the virtual value of output voltage is directly proportional to the synthesis of angular rate Ω of rotating carrier between yaw and pitch.

**Figure 2.**
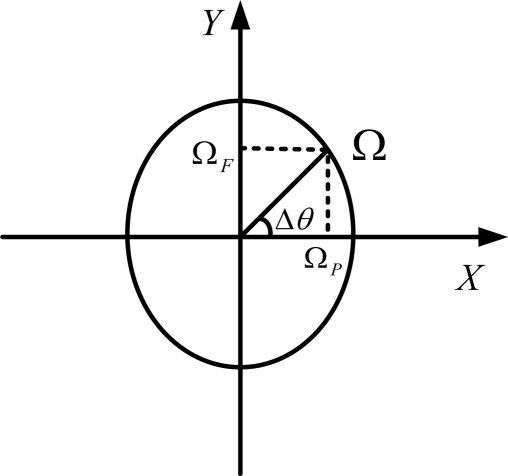
The relationships between Ω, Ω*_P_*,Ω*_F_*, and Δ*θ*.

In order to obtain yaw and pitch angular rate, an accelerometer is used, which is attached to the rotating carrier. The output of the accelerometer is:

(3) $$u_{2}=A\text{sin}(\dot{\phi }t+\theta_{2})$$

where *A* is output max value of output, which depends on the rolling rate *φ̇*, *θ*_2_ is the initial phase, The frequency of the output signal is the same as the rotating carrier’s rolling frequency. According to the study above, the synthesis angular rate of the rotating carrier is determined by measuring the phase difference between the signals of gyroscope and accelerometer. The relationships between Ω, Ω*_P_*,Ω*_F_*, and Δ*θ* = *θ*_1_ (*t*) – *θ*_2_ are shown in Figure 2.

Thus we can obtain the following expressions:

(4) $$\Omega_{P}=U_{1}\;\text{cos}(\theta_{1}(t)\;-\theta_{2})/k=U_{1}\text{cos}(\Delta \theta )/k$$

(5) $$\Omega_{F}=U_{1}\text{sin}(\theta_{1}(t)-\theta_{2})/k=U_{1}\;\text{sin}(\Delta \theta )/k$$

Here, Ω*_F_* is pitch angular rate, Ω*_P_* yaw angular rate, and *U*_1_ the virtual value of *u*_1_.

## 3. Digital Circuits Design

### 3.1. Hardware Circuits Design

**Figure 3.**
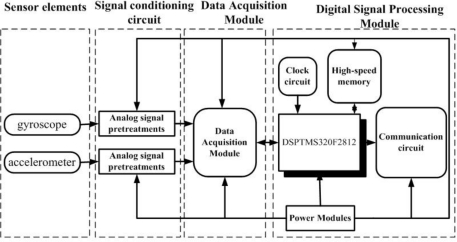
The signal-conditioning circuit diagram.

In order to implement all of the above-mentioned operations, we have designed signal-conditioning hardware circuits as shown in Figure 3. The hardware circuits consist of four parts: sensing elements, analog signals pretreatments, data acquisition module and signal demodulation processing circuits.

**Figure 4.**
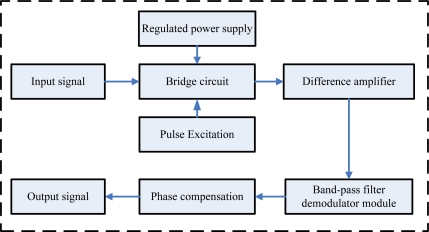
The gyroscope signal pretreatment circuit.

**Figure 5.**
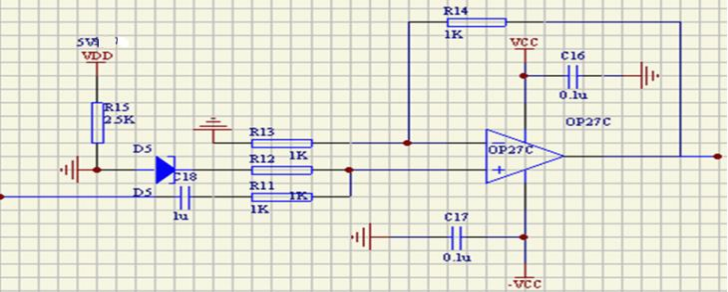
The accelerometer signal pretreatment circuit.

The first part includes two sensing elements: gyroscope and accelerometer, which sense the angular rate and the direction, respectively. The second part is used to deal with the analog signals of the sensing elements, including the gyroscope signal pretreatment circuit and the accelerometer pretreatment circuit, shown in Figure 4 and Figure 5, respectively. The gyroscope signal pretreatment circuit mainly consists of the regulated power supply, bridge circuit, pulse excitation, difference amplifier, band-pass filter demodulator and phase compensation, *etc*. It is capable of complete detection of small capacitances and transferring the capacitance into a voltage signal that ranges from −10 V to +10 V.

The output signal of the accelerometer contains DC component, which changes with different rotating rates. In addition to this, the output AC component of the accelerometer is weak, about −0.25 V∼+0.25V. To solve these problems, we use a capacitance (c18) to filter out the DC, then make up the output signal though an operational amplifier circuit to amplify it 10 times, coupled with a superimposed voltage of 2.5 V DC, so the output voltage is in the range 0 V∼5 V.

**Figure 6.**
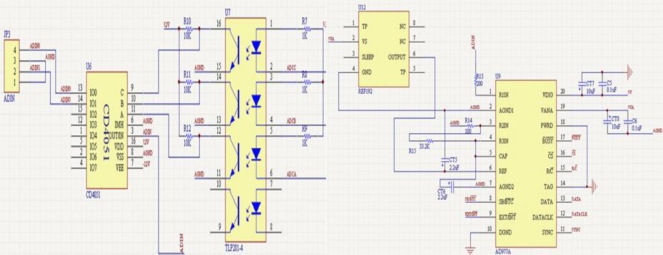
The data acquisition circuit.

The third part is data acquisition module as shown in Figure 6, which mainly consists of AD977A, CD4051 and TLP281-4. There are two input signals but AD977A only has one input port, so we designed a switch module. The CD4051 analog multiplexer is a digitally-controlled analog switch having low ON impedance and very low OFF leakage current; it is a single 8-channel multiplexer having three binary control inputs, A, B and C, and an inhibit input. The three binary signals select 1 of 8 channels to be turned on, and connect one of the 8 inputs with the output. A, B and C are controlled by TLP281-4 alternately to sample the gyroscope and accelerometer signals.

The last part is the signal demodulation processing circuits based on DSP2812 and peripheral circuits. The peripheral circuits include power module, reset circuit and serial port communication circuit of DSP2812, *etc*. We use a MAX3232 chip as communication for signal output. The 5V power design is based on the LM2576 and the 1.8 V and 3.3 V voltage is transferred by using the LM1117.

### 3.2. Signal Processing Algorithm

**Figure 7.**
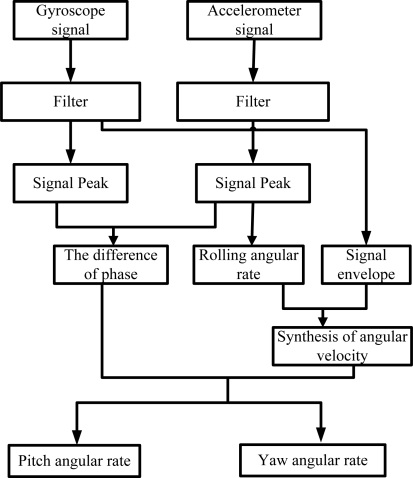
The signal-conditioning processing.

The signal processing is shown in Figure 7. First, we can filter the signal and calculate the frequency of the sensing element (accelerometer) signal, which is same as the rotating carrier rolling rate; Secondly, we calculate the peak output voltage of the signals and get the phase difference between gyroscope and accelerometer. At the same time, we can obtain the envelop of the gyroscope signal, which is proportional to the fused angle rate of yaw and pitch. According to the phase difference between gyroscope signal and accelerometer signal, we can obtain yaw angular and pitch angular rate.

## 4. Test Results

**Figure 8.**
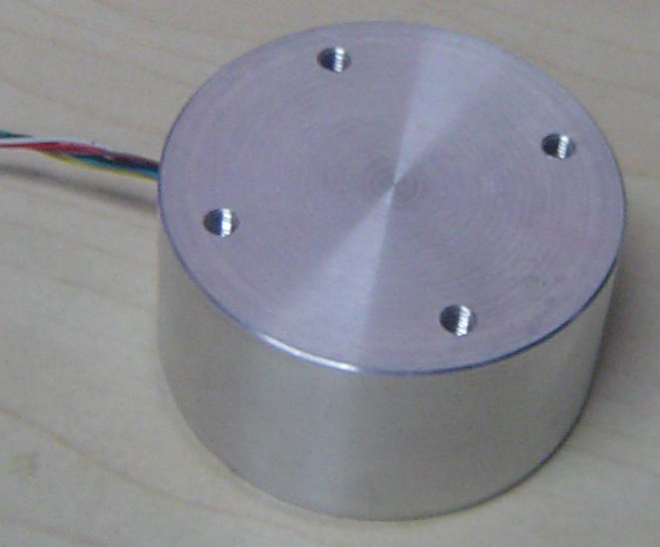
The appearance of a new digital angular rate sensor.

**Figure 9.**
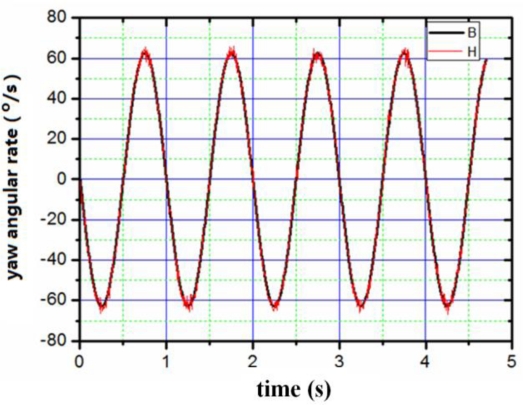
Comparison between the input yaw angular rate and the output yaw angular rate. B: the input of yaw angular rate; H: the output of yaw angular rate.

**Figure 10.**
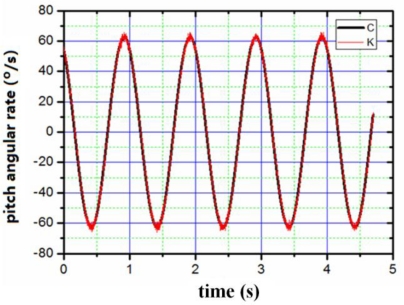
Comparison between the input of pitch angular rate and the output of pitch angular rate. C: the input of yaw angular rate; K: the output of yaw angular rate.

**Figure 11.**
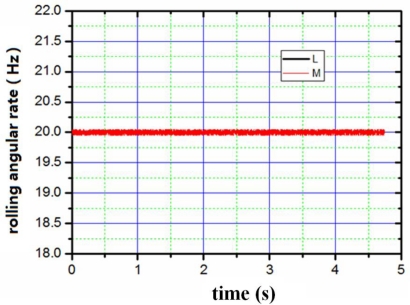
Comparison between the input of rolling angular rate and output of rolling angular rate. L: the input of rolling angular rate; M: the output of rolling angular rate.

The appearance of the digital angular rate sensor is shown in Figure 8. The device has the shape of a cylinder with a diameter of 5 cm and a height of 4 cm. The sensor can be mounted on a simulator of a rotating carrier system designed specifically to apply this sensor test. The system consists basically of a three-axis servo table. One is used to simulate the rotating carrier, and the others two control the attitude of the rotating carrier. The simulator rolls as a rotating carrier with yaw and pitch angular rate. The digital outputs are recorded by a computer. First of all, we set a rolling rate at 20 Hz, then set yaw and pitch angular vibration frequency as 1 Hz, vibration amplitude as 10°, respectively. The output results were recorded by the computer. Figures 9–11 illustrate the comparison between the measured angular rate and the actual rate.

The digital angular rate sensor can sense rolling, yaw and pitch angular rate of rotating carrier at one time. From Figures 9–11, it is clear that the measurement results match the actual results very well. The relative error of the sensor is less than 0.5%.

## 5. Conclusions

The construction, operation principles, circuits, signals processing and tests results of a novel digital angular rate sensor are described. The sensor, which has the advantages of simple structure, small size and low cost, can sense rolling rate, yaw angular rate and pitch angular rate. The experiments show that the measured angular rate (rolling, yaw and pitch angular rate) agree with the actual rate in cases of different angular rate and the max error is less than 0.5%. The effectiveness of the sensor is validated.
